# Enhanced capacitive deionization performance using Mn-doped activated carbon electrodes for energy-efficient brackish water desalination

**DOI:** 10.1098/rsos.250372

**Published:** 2025-06-18

**Authors:** Nasser A. M. Barakat, Eman Ashour, Yasmin T. Sayed

**Affiliations:** ^1^ Chemical Engineering Department, Minia University, Minya, Egypt

**Keywords:** capacitive deionization, Mn-doped activated carbon, water desalination, electrosorption, energy-efficient electrodes

## Abstract

Capacitive deionization (CDI) has emerged as a promising alternative for brackish water desalination due to its low energy consumption and operational simplicity. However, the performance of CDI is highly dependent on the properties of the electrode materials. In this study, Mn-doped activated carbon (Mn-AC) electrodes were synthesized and evaluated for enhanced ion removal efficiency in CDI systems. The Mn doping process was optimized using hydrothermal synthesis with varying KMnO_4_ precursor concentrations. Structural characterization via X-ray diffraction, Fourier transform infrared, scanning electron microscopy and elemental mapping confirmed successful Mn incorporation, while thermogravimetric analysis demonstrated improved thermal stability. Electrochemical studies, including cyclic voltammetry and chronoamperometry, revealed that Mn-AC electrodes exhibited higher specific capacitance and superior ion adsorption capacity compared with pristine activated carbon. The CDI performance was evaluated at different applied voltages and NaCl concentrations, demonstrating a significant increase in electrosorption capacity with optimized Mn doping. The highest electrosorption capacity was achieved at +1.2 V with 0.1 M NaCl, where Mn-AC exhibited a 33% higher adsorption efficiency than pristine AC. These findings highlight the potential of Mn-AC as an efficient electrode material for high-performance CDI applications, providing a sustainable and scalable solution for water desalination.

## 1. Introduction

Water scarcity is one of the most pressing global challenges of the twenty-first century, affecting billions of people worldwide. Rapid population growth, industrialization and climate change have exacerbated the demand for fresh water, while natural freshwater resources are becoming increasingly depleted [1]. To address this crisis, desalination technologies have emerged as a critical solution for producing fresh water from saline sources, such as seawater and brackish water. However, conventional desalination methods, such as reverse osmosis and thermal distillation, are energy-intensive and costly, limiting their applicability in resource-constrained regions [2]. As a result, there is a growing need for low-energy, cost-effective desalination technologies that can provide sustainable access to clean water.

Among the emerging desalination technologies, electrochemical methods have gained significant attention due to their low energy consumption, scalability and environmental friendliness. These methods include electrodialysis, membrane capacitive deionization and capacitive deionization (CDI). CDI, in particular, has emerged as a promising technology for brackish water desalination due to its simplicity, low operational costs and ability to operate at ambient conditions [3]. CDI works by applying an electric potential across two porous electrodes, typically made of carbon materials, to adsorb ions from water through electrostatic attraction. When the electric field is removed or reversed, the ions are released, and the electrodes are regenerated for subsequent cycles [4].

Despite its advantages, the widespread application of CDI faces several challenges, particularly related to the performance and cost of electrode materials. The electrodes are the most critical component of CDI systems, as they determine the ion adsorption capacity, charge efficiency and long-term stability of the system. Conventional CDI electrodes are typically fabricated from carbon-based materials, such as activated carbon (AC), carbon nanotubes and graphene, due to their high surface area, electrical conductivity and chemical stability [5]. However, these materials often suffer from limited ion adsorption capacity and poor selectivity, which restrict their desalination performance.

To overcome these limitations, researchers have explored the use of transition metal-based compounds as electrode materials, owing to their high redox activity, excellent ion adsorption properties and tunable surface chemistry. Among these, manganese-based compounds have shown great promise due to their abundance, low cost and environmental compatibility [6]. For example, manganese oxides (e.g. MnO₂, Mn₃O₄) have been widely studied for their high pseudocapacitive behaviour, which enhances ion adsorption capacity and improves the overall desalination performance of CDI electrodes [7]. Additionally, Mn-based compounds exhibit excellent electrochemical stability and can be easily integrated into carbon-based electrodes.

AC has been widely used as a support material for Mn-based compounds due to its high surface area, porous structure and excellent electrical conductivity. The combination of Mn compounds with AC not only enhances the electrochemical performance of the electrodes but also provides a cost-effective solution for large-scale CDI applications [8]. For instance, MnO₂–AC composites have demonstrated superior desalination performance compared with pure AC electrodes, owing to the synergistic effects of MnO₂’s redox activity and AC’s conductive network [9]. Similarly, Mn₃O₄–AC composites have shown enhanced ion adsorption capacity and cycling stability, making them attractive candidates for CDI electrodes [10].

In this study, we propose a novel amorphous Mn-doped AC (Mn-AC) material as an electrode for CDI applications. The incorporation of Mn into the AC matrix is expected to enhance the ion adsorption capacity, charge efficiency and cycling stability of the electrode, while maintaining the low-cost and scalable nature of AC. The hydrothermal synthesis method used in this study ensures a uniform distribution of Mn species on the AC surface, which is critical for achieving high desalination performance.

The proposed Mn-AC material offers several advantages over conventional CDI electrodes. First, the high surface area and porous structure of AC provide ample sites for ion adsorption, while the Mn species enhance the electrochemical activity of the electrode. Second, the low-cost and abundant nature of both AC and Mn precursors make this material economically viable for large-scale applications. Finally, the hydrothermal synthesis method used in this study is simple, scalable and environmentally friendly, making it suitable for industrial production. By addressing the limitations of conventional CDI electrodes, this study aims to contribute to the development of low-energy, cost-effective desalination technologies that can provide sustainable access to clean water.

## 2. Experimental section

### 2.1. Materials

The synthesis of Mn-AC required commercially available AC and potassium permanganate (KMnO₄, greater than or equal to 99%, Sigma-Aldrich) as the Mn precursor. Deionized (DI) water was used for all preparation and washing steps. For the electrochemical measurements, Nafion solution (5 wt% in isopropanol, Sigma-Aldrich) and isopropanol (greater than or equal to 99.5%, Sigma-Aldrich) were utilized. In the fabrication of CDI electrodes, carbon cloth (3.5 × 3.5 cm, commercial grade) was used as the current collector, while polyvinylidene fluoride (PVDF, ≥99%, Sigma-Aldrich) served as the polymeric binder, with N,N-dimethylformamide (DMF, greater than or equal to 99.8%, Sigma-Aldrich) as the solvent.

### 2.2. Synthesis of Mn-doped activated carbon

The Mn-AC was synthesized using a hydrothermal method in a Teflon-lined stainless-steel reactor with a capacity of 200 ml. Different samples were prepared by varying the weight percentage of KMnO_4_ relative to AC (0, 0.7, 1, 2, 5 and 10 wt%). In each experiment, 1 g of AC was dispersed in 100 ml of DI water, and the appropriate amount of KMnO_4_ was added while stirring to ensure uniform distribution. The mixture was then transferred into the hydrothermal reactor and heated at 180°C for 10 h. After the reaction, the product was filtered, washed thoroughly with DI water and dried at 100°C for 2 h to obtain Mn-AC with varying Mn contents.

In the hydrothermal synthesis process, the reaction was carried out in a sealed Teflon-lined stainless-steel autoclave, and the temperature was maintained at 180°C. Under such conditions, water exists in a subcritical state, meaning it is above its standard boiling point (100°C) but below the critical temperature (374°C). According to standard steam tables, the corresponding saturated vapour pressure of water at 180°C is approximately 1002.7  kPa (10.03  bar). In this state, water exhibits unique properties such as reduced dielectric constant and enhanced ion product, which facilitate the dissolution and redox activity of ionic species, such as KMnO_4_. These properties make subcritical water an efficient medium for promoting surface modification and doping reactions on AC substrates.

Subcritical water exhibits unique physico-chemical properties, including reduced dielectric constant, enhanced ion product and increased diffusivity, that make it an excellent medium for chemical reactions and material synthesis [11]. Under these conditions, the dissociation of potassium permanganate is favoured, leading to the formation of Mn²^+^ and Mn³^+^ ions rather than crystalline manganese oxides. The high ion product of subcritical water promotes the hydrolysis of KMnO_4_, while the reduced dielectric constant facilitates the dispersion of Mn species onto the AC surface [12]. This process ensures that the Mn atoms are uniformly doped into the AC matrix, rather than forming discrete crystalline phases such as MnO_2_ or Mn_3_O_4_. Furthermore, the hydrothermal treatment at 180°C allows for the functionalization of the AC surface with oxygen-containing groups, which can act as anchoring sites for Mn ions, enhancing their stability and distribution [13].

### 2.3. Fabrication of capacitive deionization electrodes

The CDI electrodes were fabricated by preparing a polymeric ink. Initially, 1 g of PVDF was dissolved in 8 ml of DMF under continuous stirring until a homogeneous binder solution was obtained. Then, 0.5 g of either pristine or Mn-AC was added to the solution, forming a uniform ink. This ink was coated onto carbon cloth electrodes (3.5 × 3.5 cm) using a blade-coating technique. The coated electrodes were first dried at 60°C for 5 h to remove excess solvent. Subsequently, they were subjected to heat treatment in a tube furnace with a controlled heating rate of 2°C min^−1^ and a final holding temperature of 350°C for 3 h to enhance their stability and electrochemical performance.

### 2.4. Characterizations

The synthesized materials were characterized using multiple analytical techniques. X-ray diffraction (XRD) was performed using a Rigaku Ultima IV diffractometer with Cu Kα radiation (*λ* = 1.5406 Å), scanning over a 2*θ* range of 10°−80° at a step size of 0.02° s^−1^. The surface morphology and elemental composition were analysed using FEI Quanta 250 FEG scanning electron microscopy (SEM), equipped with energy-dispersive X-ray spectroscopy for elemental mapping. Fourier transform infrared (FTIR) spectra were recorded using a Bruker Vertex 70 FTIR spectrometer in attenuated total reflectance mode within a spectral range of 4000−400 cm^−1^. Thermogravimetric analysis (TGA) was conducted using a TA Instruments Q500 TGA under a nitrogen atmosphere, with a heating rate of 10°C min^−1^ up to 900°C to assess the thermal stability of the samples.

### 2.5. Electrochemical measurements

**Figure 1.**
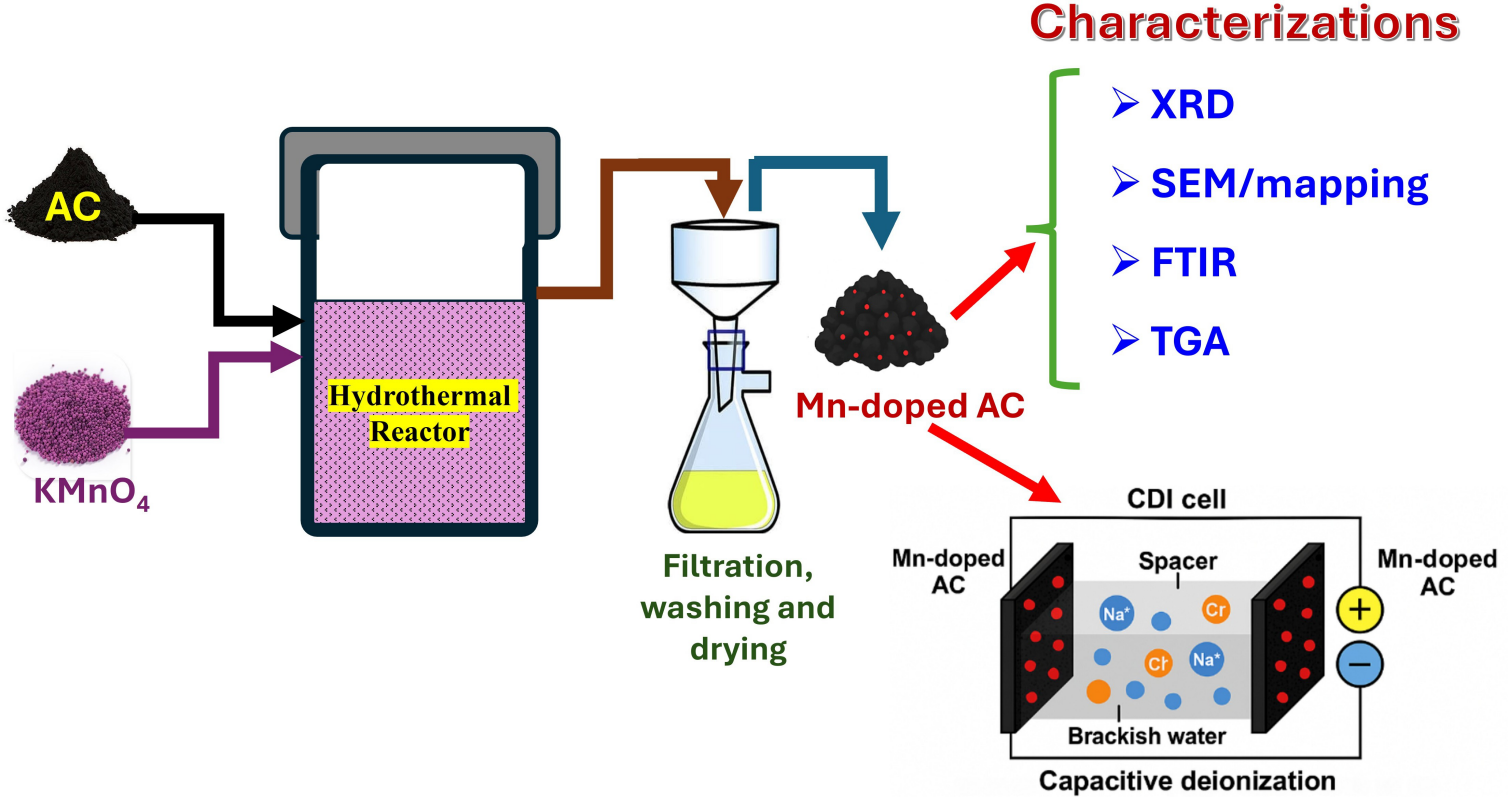
Schematic illustration of the synthesis, characterization and CDI application of Mn-doped activated carbon electrodes.

Cyclic voltammetry (CV) measurements were conducted using a three-electrode electrochemical cell. The working electrode was prepared by modifying a glassy carbon electrode with the synthesized material. An Ag/AgCl (saturated KCl) electrode served as the reference electrode, while a graphite rod was used as the counter electrode. The working electrode was fabricated by dispersing 0.002 g of the functional material in 20 µl of Nafion solution and 400 µl of isopropanol. The dispersion was ultrasonicated to ensure homogeneity, and the ink was drop-cast onto the glassy carbon electrode in three successive layers, with each layer consisting of 5 µl of ink. After each deposition, the electrode was allowed to dry naturally before applying the next layer. Following the final deposition, the electrode was dried at 80°C for 30 min. All electrochemical measurements were carried out using an Autolab PGSTAT302N potentiostat in different concentrations of NaCl electrolyte solutions. The CV scans were performed within a potential window of −0.2 to 0.8 V versus Ag/AgCl at scan rates ranging from 5 to 100 mV s^−1^. This procedure ensured a comprehensive assessment of the electrochemical behaviour and capacitive performance of the Mn-AC electrodes for CDI applications. All electrochemical measurements, including CV and chronoamperometry, were performed in triplicate to ensure the reliability of the data. The results are presented as mean values with standard deviations, and error bars representing these standard deviations have been included in the relevant figures. The observed variability across repeated measurements was within ±5% of the reported values, indicating high reproducibility of the experimental procedures. Figure 1 shows schematic diagram for the synthesis, characterization and CDI application of Mn-AC electrodes.

## 3. Results and discussion

### 3.1. Electrode characterization

**Figure 2.**
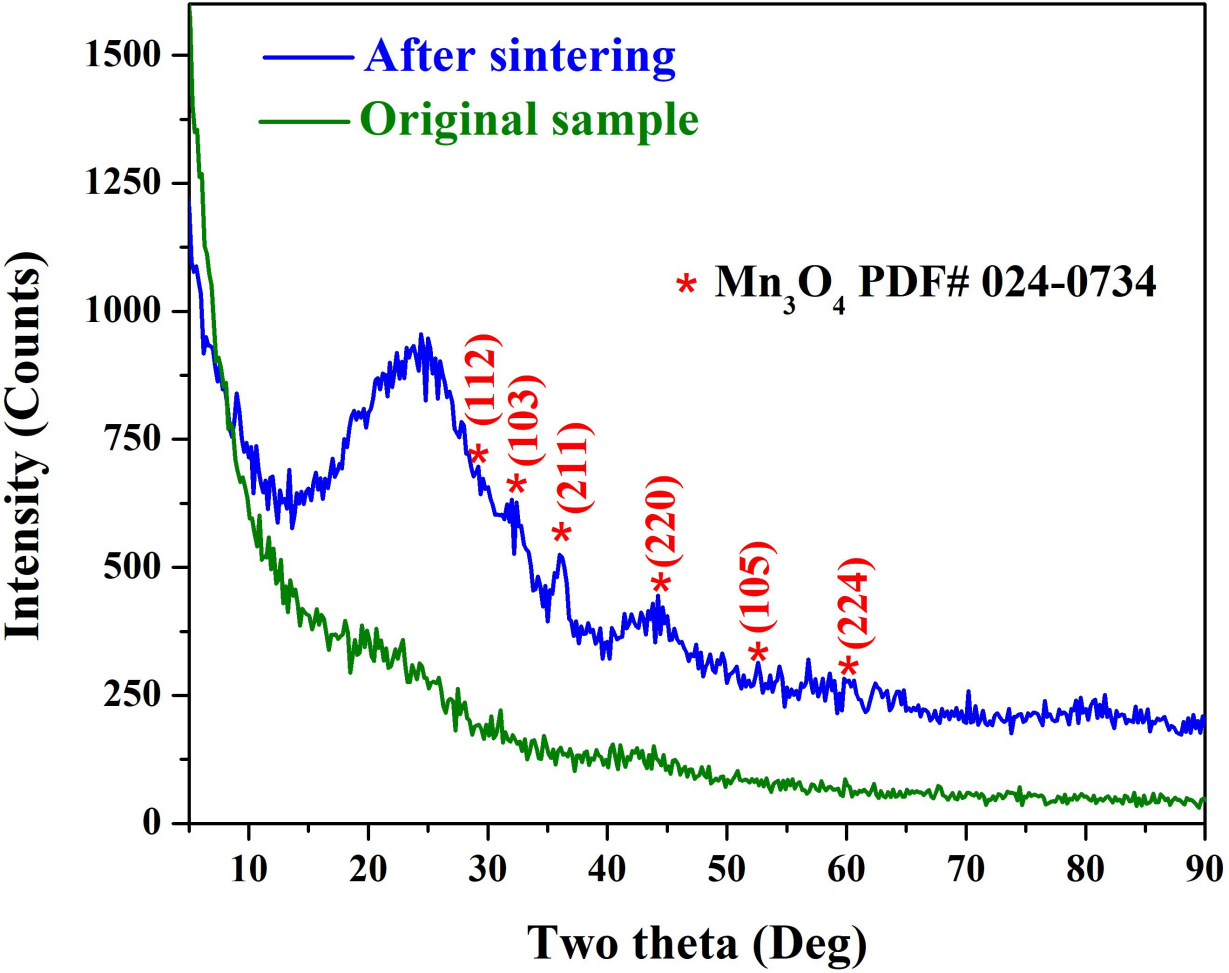
XRD patterns for the as-obtained hydrothermally treated (0.02 g KMnO_4_ sample) AC and after sintering in inert atmosphere at 700°C for 2 h.

The structural and phase composition of the synthesized Mn-AC was investigated using XRD analysis (figure 2). The goal of this characterization was to confirm the successful doping of manganese into the AC matrix and to understand the phase transformation upon thermal treatment.

As shown in the figure, the XRD pattern of the as-synthesized Mn-AC sample did not exhibit any distinct diffraction peaks corresponding to manganese-containing compounds. However, upon sintering the sample at 700°C for 2 h under an inert atmosphere, significant changes were observed in the XRD pattern. The heat treatment resulted in the appearance of distinct peaks corresponding to Mn_3_O_4_ (Hausmannite phase), as confirmed by comparison with ICCD# 024−0734.

The absence of Mn-representative peaks in the hydrothermally treated sample may indicate that the manganese species were either present in an amorphous state or well-dispersed within the carbon matrix in atomic form, preventing their detection via XRD. When metal species are present as nano-sized clusters, their diffraction peaks may exhibit significant broadening and reduced intensity due to the small coherent scattering domains and increased structural disorder, which can make detection and precise identification challenging. Moreover, the interaction between manganese species and the porous carbon framework during the hydrothermal synthesis may have resulted in an amorphous phase, lacking long-range crystalline order. Furthermore, manganese ions may have formed interactions with oxygen-containing functional groups present on the AC surface, preventing the formation of crystalline Mn compounds [13,14].

The emergence of Mn_3_O_4_ diffraction peaks suggests that thermal activation facilitated the nucleation and growth of manganese oxide crystallites, leading to phase transformation. During hydrothermal synthesis, manganese from potassium permanganate may have been incorporated into the carbon structure in an amorphous state. High-temperature sintering enables partial oxidation and rearrangement of manganese species, leading to Mn_3_O_4_ crystallization. The ability of elevated temperatures to promote the migration and transformation of manganese species has been demonstrated in previous studies. Ashoka *et al*. reported that KMnO_4_ undergoes reduction to form Mn_3_O_4_ through a hydrothermal process at 180°C, confirming that such thermal conditions provide sufficient energy to drive redox reactions and facilitate manganese mobility in solution [15]. Similarly, Becerra *et al*. showed that the thermal decomposition of KMnO₄ at elevated temperatures leads to the formation of manganese oxide phases with enhanced catalytic properties, indicating the role of heat in enabling structural rearrangements and the redistribution of Mn species within the solid matrix [16]. These findings support the mechanism proposed in this work, where the hydrothermal treatment allows for uniform Mn incorporation onto the AC surface.

A potential concern is whether potassium permanganate was merely adsorbed onto the AC surface during the hydrothermal treatment and later decomposed during the sintering process. However, this claim is not valid for several reasons: if KMnO_4_ had been physically adsorbed onto the AC surface, its characteristic diffraction peaks would have been detectable in the XRD pattern of the as-synthesized sample. The lack of such peaks strongly indicates that KMnO_4_ did not remain in a crystalline state within the carbon matrix after hydrothermal treatment [17]. After hydrothermal treatment, the sample was thoroughly washed with DI water to remove any residual KMnO_4_. This step ensures that only chemically incorporated or transformed manganese species remain within the carbon framework, preventing any surface-adsorbed KMnO_4_ from interfering with subsequent thermal transformations. Moreover, KMnO_4_ is highly reactive under hydrothermal conditions, and its reaction with carbon-based materials typically leads to the formation of MnO*x* species rather than simple surface adsorption. The oxidative environment promotes the integration of Mn species into the carbon structure, reducing the likelihood of physical adsorption without chemical interaction.

The broad peak observed around 25° 2*θ* is indicative of the amorphous nature of the carbon material. This peak corresponds to the (002) plane of graphitic carbon, reflecting the average interlayer spacing between carbon sheets. Using Bragg’s law (*nλ = 2 d* sin*θ*), with Cu Kα radiation (*λ* = 1.5406 Å), the interlayer spacing (*d*) is calculated as follows:


(3.1) d=λ/(2sinθ)=1.5406 Å/(2sin25°)≈1.78 Å.

However, this value seems inconsistent with typical interlayer spacings reported for amorphous carbon. Previous studies have reported interlayer spacings for amorphous carbon materials to be approximately 3.56 Å, which is larger than the interlayer spacing in crystalline graphite (3.35 Å) due to the disordered stacking of graphene layers. This discrepancy suggests that the broad peak at 25° 2*θ* in our XRD pattern may be influenced by factors such as instrumental broadening or the presence of other structural features. Therefore, the observed peak at 25° 2*θ* is characteristic of the turbostratic structure commonly found in disordered carbon materials.

**Figure 3.**
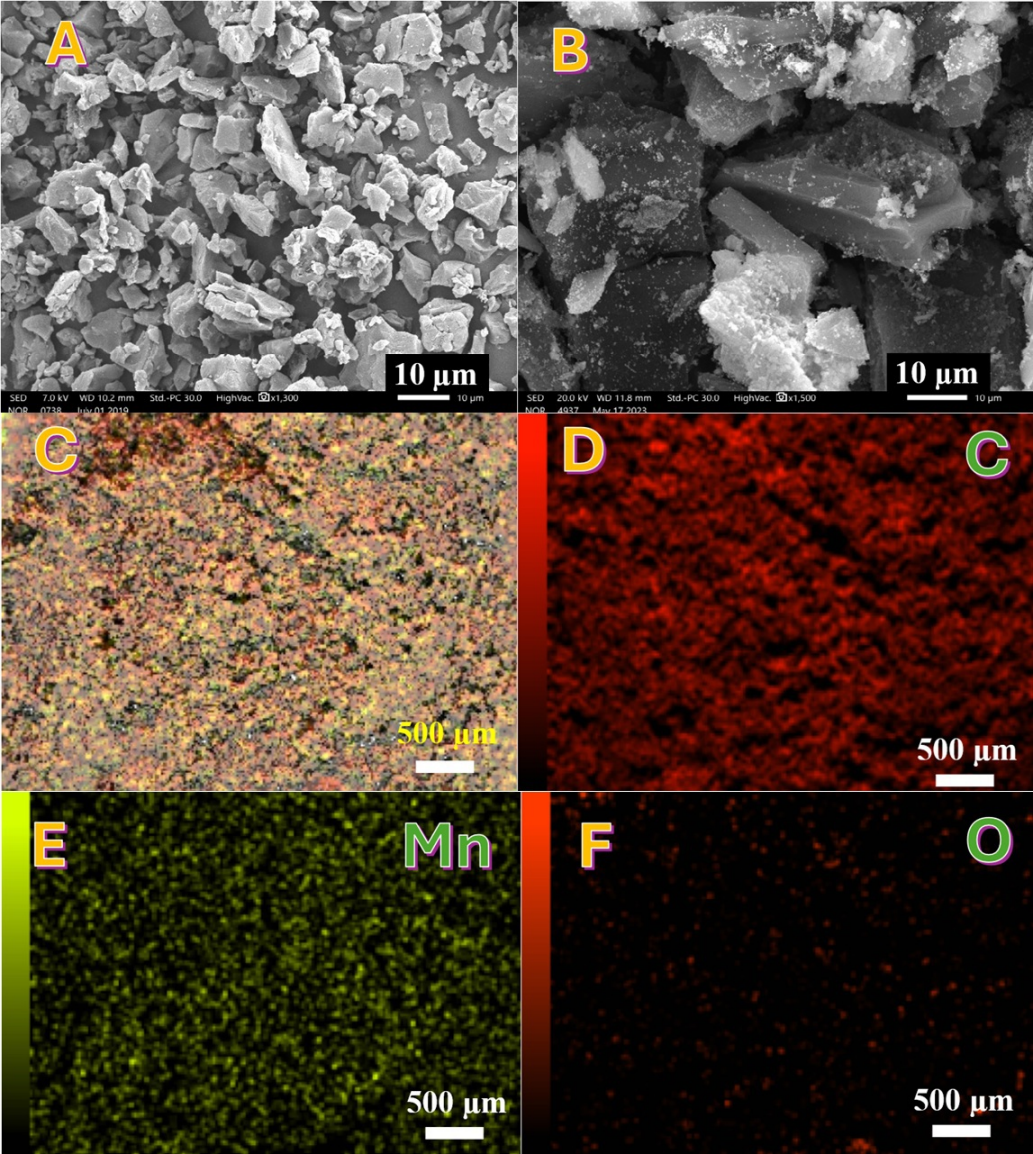
(A,B) SEM images of the Mn-doped activated carbon sample (hydrothermally treated but not sintered) at two different magnifications. (C) Elemental mapping overview image of the same sample. (D–F) Elemental distribution maps for carbon (D), manganese (E) and oxygen (F).

The SEM images presented in figure 3, panel A reveals the morphology of the Mn-AC, while, panel B shows the untreated AC at the same magnification. As shown, the average particle size of the treated AC was found to be approximately 3 μm, which is significantly smaller than that of the untreated AC. The observed size reduction suggests that hydrothermal treatment led to partial fragmentation and restructuring of the carbon framework. This phenomenon can be attributed to the reaction between KMnO_4_ and AC probably facilitated surface oxidation and removal of weaker carbon domains, leading to size reduction. The hydrothermal conditions may have induced pore development, which further contributed to particle disintegration. Moreover, the elevated temperature and pressure during hydrothermal synthesis could have disrupted the larger carbon particles, breaking them down into smaller fragments [18].

Elemental mapping was performed to further confirm the presence and distribution of manganese on the surface of the hydrothermally treated AC. This technique provides spatial resolution of elemental composition, ensuring that Mn incorporation occurred successfully. Panel C illustrates the elemental distribution of all detected elements (C, O, Mn), confirming the uniform dispersion of Mn throughout the AC surface. Panel D presents the elemental mapping of carbon, showing a high-intensity colour due to the predominant presence of carbon within the AC structure. Panel E displays the manganese distribution, where a relatively lower intensity compared with carbon was observed. This confirms the successful incorporation of Mn into the AC matrix, supporting the claim that Mn is chemically integrated rather than simply adsorbed. Panel F demonstrates the oxygen distribution, where surprisingly low intensity was recorded compared with Mn and C. The relatively low oxygen content observed in the elemental mapping data may be explained by several factors: (i) reduction of oxygen functional groups during hydrothermal treatment, the interaction between KMnO_4_ and the AC surface probably resulted in oxidation reactions, leading to the loss of oxygen-containing functional groups; (ii) preferential Mn-O bond formation: manganese species may have selectively bonded with oxygen sites on the carbon surface, reducing the availability of free oxygen; and (iii) surface carbonization effects: the high-temperature conditions during drying and hydrothermal synthesis may have led to mild carbonization, further decreasing the surface oxygen content.

**Figure 4.**
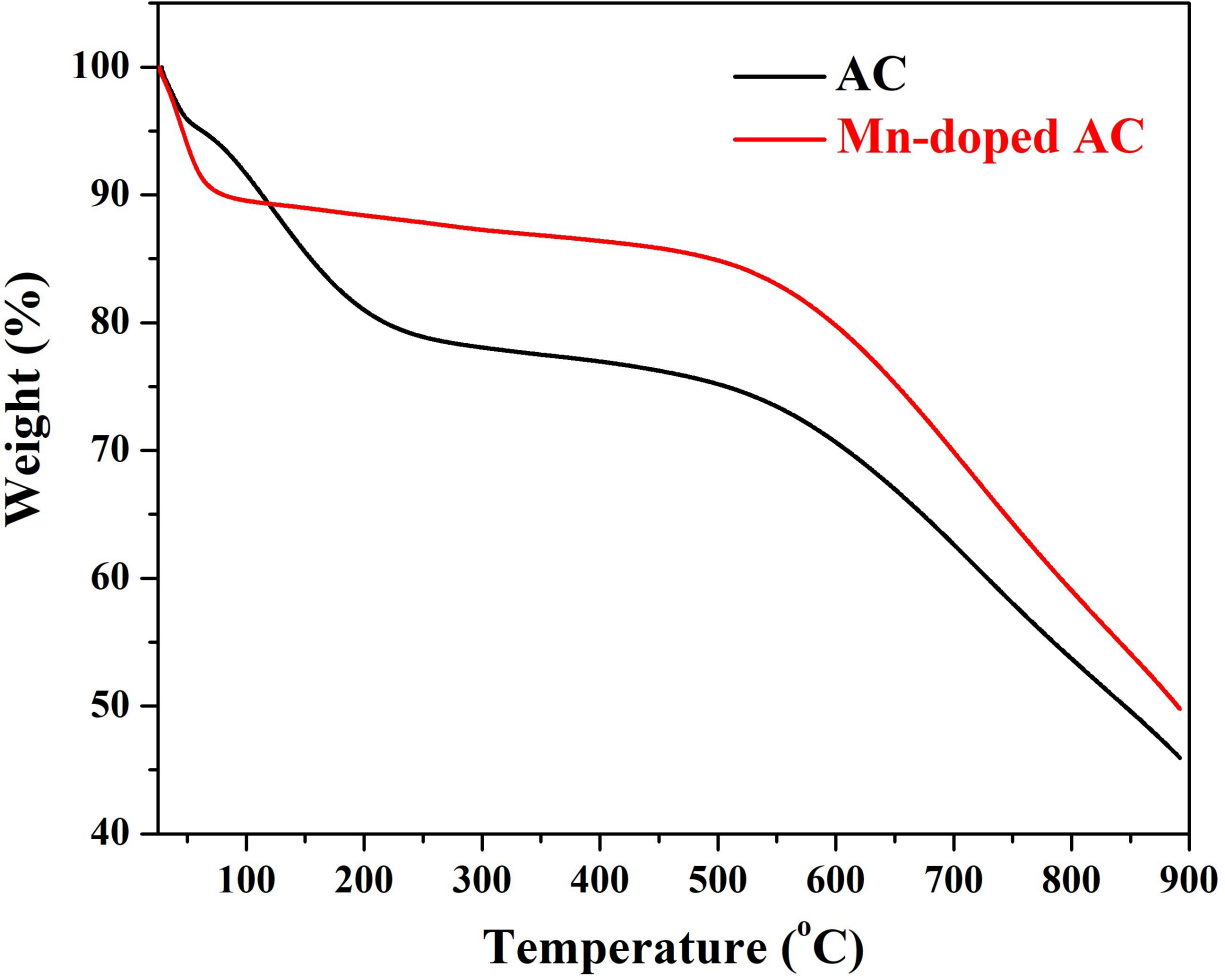
TGA analyses for the pristine and hydrothermally treated (Mn-doped AC; 2 wt%).

TGA was performed on Mn-AC and pristine AC under nitrogen atmosphere to evaluate their thermal stability and decomposition behaviour. The data are displayed in figure 4. The comparison between these two materials provides insights into how Mn incorporation influences carbon degradation and residue formation at high temperatures.

The TGA curve of pristine AC exhibited three initial stages of weight loss, followed by a plateau and a final sharp decomposition. The first stage, ending at 51.6°C with 95.7 wt% remaining, and the second stage, ending at 95°C with 92.3 wt% remaining, are attributed to the removal of physically adsorbed water and volatile organic compounds from the AC surface [19]. The third stage, ending at 220.7°C with 79.8 wt% remaining, corresponds to the oxidation of surface functional groups (e.g. carboxyl, hydroxyl and carbonyl groups) on the AC [20].

Following these initial stages, a plateau region was observed up to 502°C, with 75.15 wt% of the sample remaining. This plateau indicates a period of relative thermal stability, during which the carbon matrix resists further oxidation. At higher temperatures, a sharp weight loss occurred, with the final weight dropping to 45.92 wt% at 892°C. This final decomposition stage is attributed to the complete oxidation of the carbon matrix and the formation of gaseous products such as CO_2_ and CO [21]. The remaining residue probably consists of inorganic impurities (ash content) present in the AC, which do not oxidize under these conditions.

The TGA curve of Mn-AC showed a significantly different weight loss profile compared with pristine AC. The initial sharp weight loss, with 90.5 wt% remaining at 79°C, is attributed to the removal of adsorbed water and volatile compounds. However, the weight loss in this stage is less pronounced than in pristine AC, suggesting that the presence of Mn species may reduce the hydrophilicity of the AC surface [22].

A plateau region was observed up to 495°C, with 84.9 wt% of the sample remaining. This plateau indicates that the Mn-AC exhibits enhanced thermal stability compared with pristine AC, probably due to the interaction between the Mn species and the carbon matrix. The Mn species may act as thermal stabilizers, delaying the oxidation of the carbon matrix.

At higher temperatures, a relatively sharp weight loss occurred, with the final weight dropping to 49.78 wt% at 892°C. This weight loss is attributed to the oxidation of the carbon matrix and the formation of manganese oxides (e.g. Mn_3_O_4_ or Mn_2_O_3_). The XRD results confirmed the presence of Mn₃O₄ after sintering the sample at 700°C under an inert atmosphere, indicating that the Mn species undergo phase transformations during thermal treatment. The elemental mapping results further support this, showing a high distribution of Mn on the AC surface with relatively low oxygen content, suggesting that the Mn species are initially present in a non-crystalline or highly dispersed form.

The TGA results highlight the significant influence of Mn doping on the thermal behaviour of AC. The Mn-AC exhibits enhanced thermal stability compared with pristine AC, as evidenced by the higher remaining weight at intermediate temperatures (e.g. 84.9 wt% at 495°C for Mn-AC versus 75.15 wt% at 502°C for pristine AC). This improved stability can be attributed to the interaction between Mn species and the carbon matrix, which delays the oxidation of carbon. Additionally, the presence of Mn species may catalyse the formation of stable manganese oxides at high temperatures, contributing to the higher residual weight of Mn-AC at 892°C (49.78 wt% versus 45.92 wt% for pristine AC).

Overall, the TGA results demonstrate that Mn doping significantly enhances the thermal stability of AC, delaying the oxidation of the carbon matrix and promoting the formation of stable manganese oxides at high temperatures. These findings are consistent with the XRD and elemental mapping results, which confirm the uniform distribution of Mn species on the AC surface and their transformation into crystalline Mn_3_O_4_ upon sintering. The improved thermal stability and unique properties of Mn-AC make it a promising material for applications in CDI and other energy-related technologies.

**Figure 5.**
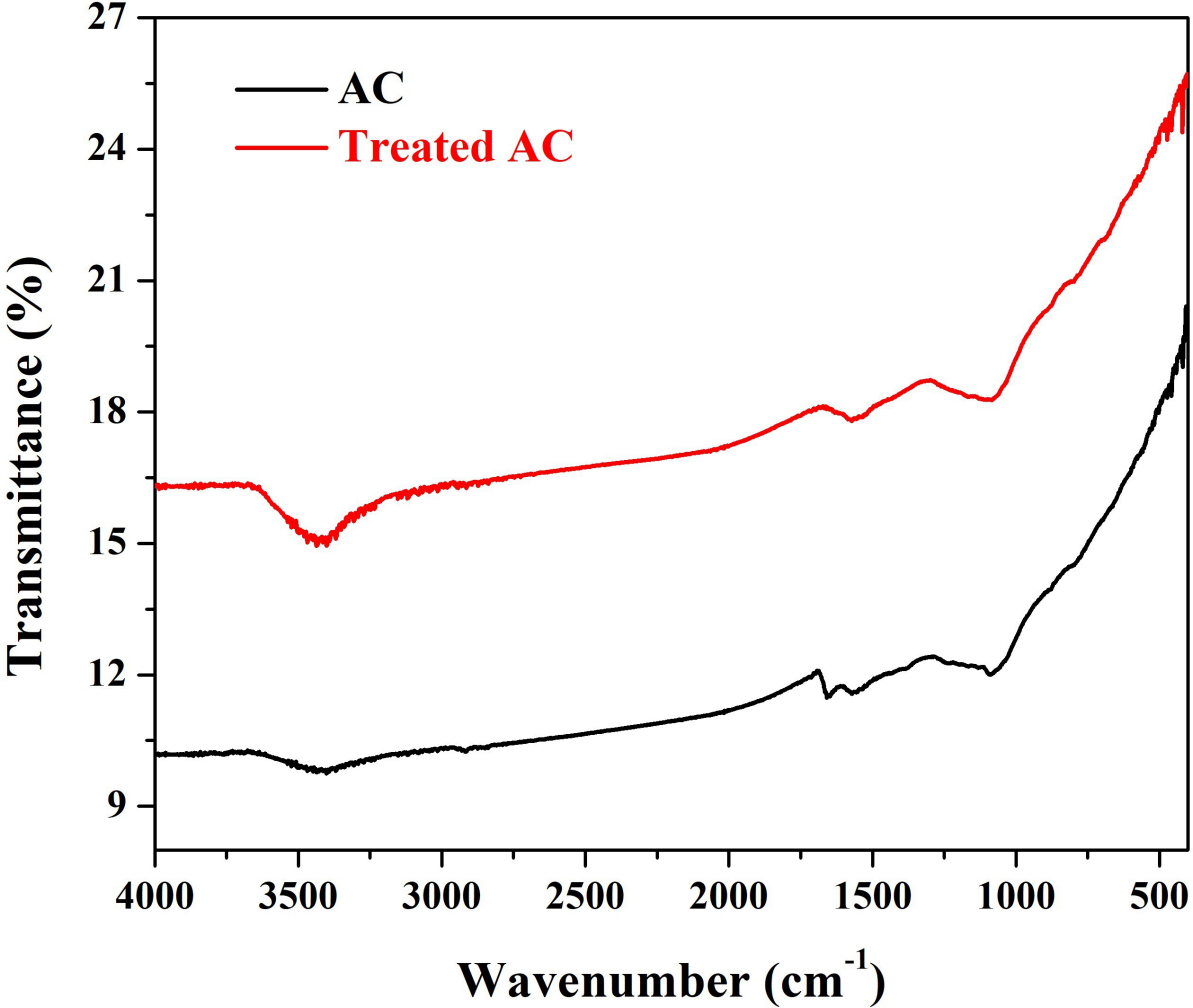
FTIR spectra for the pristine and hydrothermally treated (0.02 g KMnO_4_ sample) activated carbon.

FTIR spectroscopy was performed to analyse the functional groups present in pristine AC and Mn-AC; figure 5. This technique helps to understand how Mn incorporation affects the surface chemistry of AC by observing changes in characteristic absorption peaks. The increased intensity in the Mn-AC spectrum suggests stronger functionalization, which may be due to Mn interaction with oxygen-containing groups.

For the AC spectrum, a broad peak at approximately 3415 cm⁻¹ corresponds to the stretching vibration of hydroxyl (OH^−^) groups, probably originating from surface-bound water molecules or oxygen-containing functional groups [23]. The deepened peak, in the case of Mn-AC spectrum, suggests an increase in hydroxyl groups, which could be associated with Mn interaction with oxygen-containing species, possibly forming Mn–OH or Mn–O–C bonds.

Two small peaks at 1659 and 1565 cm⁻¹, in the case of AC sample, are associated with C=O stretching in carbonyl or quinone groups and C=C stretching vibrations in aromatic rings, respectively. Their presence indicates oxygen functionalities in the pristine AC. In the case of Mn-AC, these two peaks AC have combined into a single broad peak centred around 1565 cm⁻¹. This shift and merging indicate possible interactions between Mn and C=O/C=C functional groups, which may lead to structural reconfiguration [24].

The relatively broad peak centred at 1080 cm⁻¹ is attributed to C–O stretching vibrations from ether or carboxyl groups, which are common in AC structures. The persistence of this peak in the Mn-AC suggests that the C–O bonds are still present and relatively unaffected by Mn incorporation [25].

Both samples exhibit increasing intensity at lower wavenumbers, corresponding to vibrational modes of the carbon backbone, with potential contributions from Mn–O vibrations at lower frequencies. The rise in intensity towards 400 cm⁻¹ corresponds to skeletal vibrations of the carbonaceous material, indicating the presence of lattice distortions and structural heterogeneity.

In summary, the FTIR analysis confirms that Mn doping significantly modifies the surface chemistry of AC, leading to enhanced functionalization and restructuring of oxygen-containing groups. These changes can play a critical role in the electrochemical and adsorption properties of Mn-AC, making it a promising material for advanced applications.

### 3.2. Electrochemical measurements

#### 3.2.1. Cyclic voltammetry

**Figure 6.**
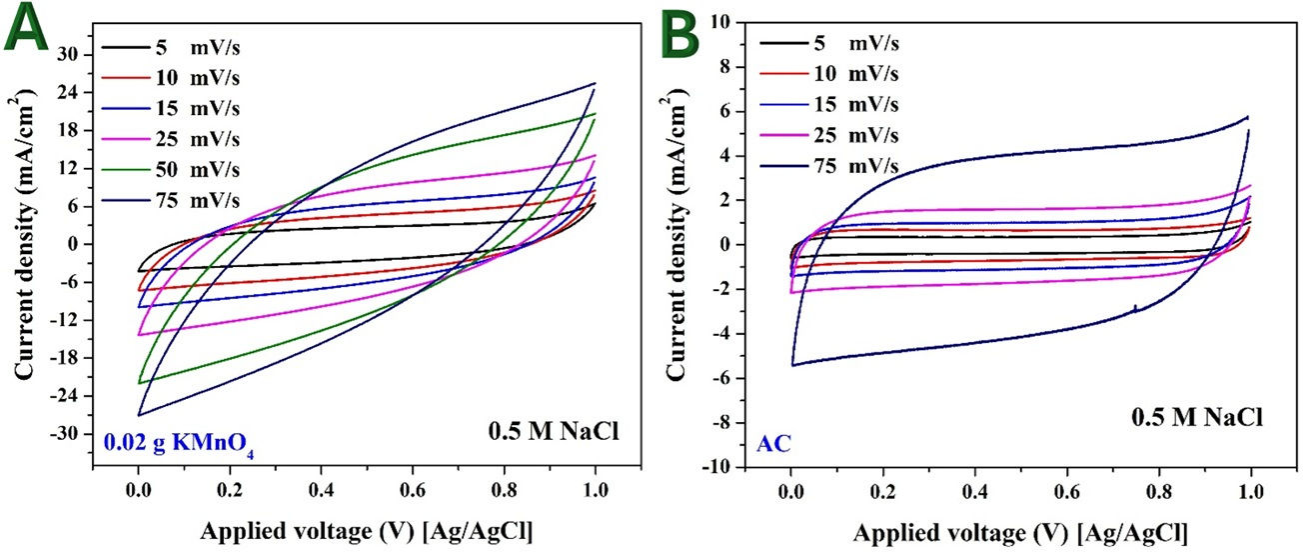
Cyclic voltammetry measurements for the prepared Mn-doped AC (2 wt% KMnO_4_ sample) (A) and pristine AC (B) electrodes in 0.5 M NaCl solution at different scan rates.

CV measurements were conducted to evaluate the specific capacitance of Mn-AC and pristine AC. The Mn-AC samples were synthesized with varying KMnO_4_ content (0.7, 1, 2, 5 and 10 wt%). The CV measurements were carried out in 0.3 and 0.5 M NaCl solutions to assess the electrochemical behaviour and potential application of these materials in CDI for brackish water desalination. As an example for the obtained measurements, figure 6 shows the obtained results for the 2 wt% Mn-doped and pristine AC electrodes in 0.5 M NaCl.

The CVs of both Mn-AC and pristine AC exhibit rectangular and symmetric shapes without distinct redox peaks. The absence of redox peaks indicates that the charge storage mechanism is predominantly electrostatic (double-layer capacitance) rather than Faradaic reactions (pseudocapacitance or battery-like behaviour) [26]. In other words, the capacitance arises from the accumulation of ions at the electrode–electrolyte interface rather than from redox reactions involving charge transfer between Mn species and the electrolyte.

This charge storage mechanism is highly desirable in the CDI cells. Electrostatic charge storage minimizes material degradation over multiple charge/discharge cycles, ensuring a long electrode lifespan in CDI applications [27,28]. Since the adsorption and desorption of ions occur at the electrode surface without slow Faradaic reactions, the process enables fast charge/discharge kinetics, making the electrodes highly efficient for water desalination. Moreover, the absence of redox processes ensures that the ion removal and regeneration steps are highly reversible, improving desalination efficiency. Finally, without redox reactions, energy loss due to Faradaic processes is reduced, resulting in lower energy consumption per desalination cycle [29].

Compared with pristine AC, the Mn-AC electrodes exhibit a higher current density in the voltammograms. Higher current density suggests greater ion adsorption capacity and improved conductivity of the electrode material. This enhancement is attributed to the presence of Mn species, which modify the carbon surface, increasing the electrochemical active surface area and improving ion transport kinetics. The higher current density correlates with an increased ability to store charge, leading to higher specific capacitance, which translates to greater salt adsorption capacity in CDI. Mn doping may introduce more functional sites or improve the surface wettability of the AC, enhancing ion mobility and reducing resistance. The incorporation of Mn may reduce charge transfer resistance, facilitating faster charge/discharge cycles and making the electrode material more suitable for high-performance CDI applications [30].

CV measurements were used to determine the specific capacitance of pristine and Mn-AC electrodes in 0.3 and 0.5 M NaCl solutions. The specific capacitance values were analysed at different scan rates, revealing trends in electrochemical performance. The specific capacitance (*C*_sp_) is derived from CV curves using the following equation [31,32]:


(3.2) $$C_{\text{sp}}=\frac{\int IdV}{2v\Delta Vm},\;$$

where *C*_sp_ is the specific capacitance (F g^−1^), *I* is the current (A), *ν* is the scan rate (V s^−1^), Δ*V* is the applied potential window (V) and *m* is the mass of active electrode materials (g). The factor 2 accounts for the charge storage in both positive and negative sweeps. By integrating the CV curve and normalizing by the scan rate, potential window and electrode mass, the specific capacitance is obtained in farads per gram (F g^−1^).

**Figure 7.**
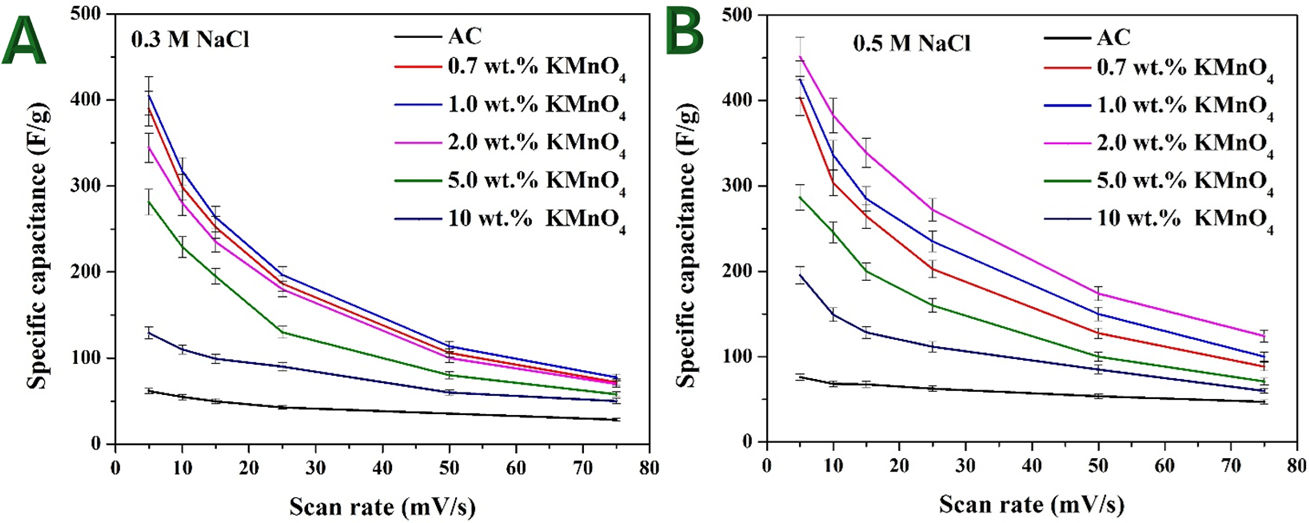
Specific capacitances of the naked and Mn-doped AC at different scan rates in 0.3 M (A) and 0.5 M (B) NaCl solutions.

For all electrodes, the specific capacitance decreases as the scan rate increases. At low scan rates, ions have sufficient time to diffuse into the electrode’s porous structure, allowing full utilization of the available surface area. At high scan rates, the diffusion of ions becomes limited due to the shorter time available for ion transport, leading to incomplete charge storage and a lower measured capacitance. As shown in figure 7, Mn-AC electrodes exhibit better capacitance retention compared with pristine AC, suggesting enhanced ionic accessibility and conductivity due to Mn incorporation [33].

**Figure 8.**
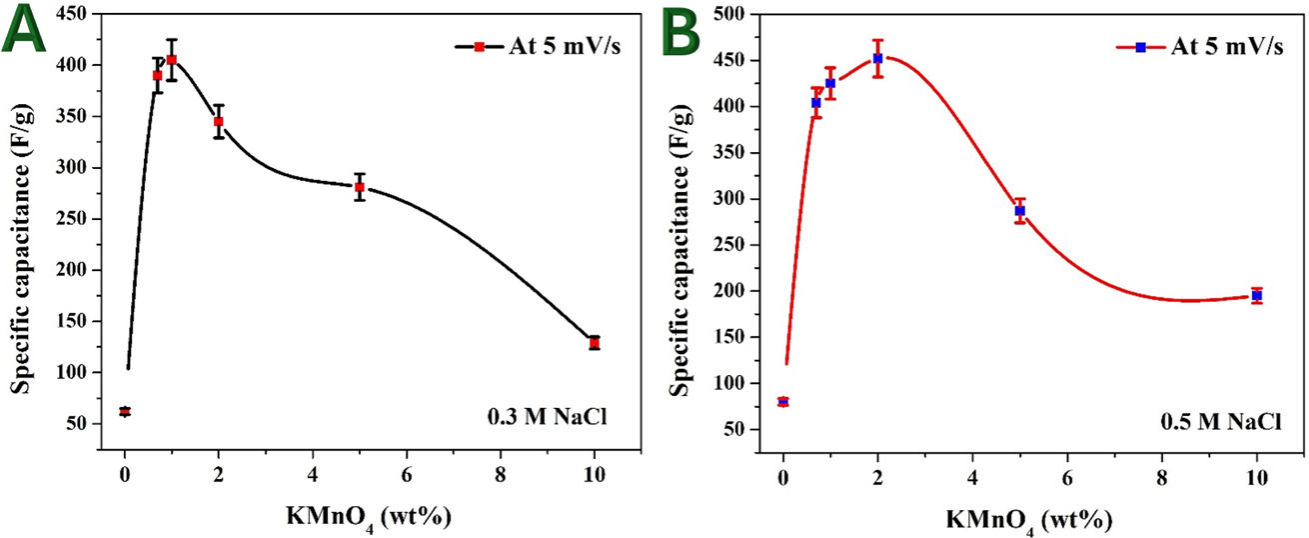
Effect of the used KMnO_4_ percentage on the specific capacitance of the prepared Mn-doped AC at scan rate 5 mV s^−1^ in 0.3 M (A) and 0.5 M (B) NaCl solution.

The relationship between KMnO_4_ percentage and specific capacitance at a scan rate of 5 mV s^−1^ was investigated in both 0.3 and 0.5 M NaCl solutions. The results are presented in figure 8A,B, respectively. These findings provide insights into the optimal Mn precursor content for maximizing the capacitive performance of Mn-AC.

The specific capacitance increases sharply from 62 F g^−1^ (pristine AC, 0 wt% Mn) to a peak value of 405 F g^−1^ at 1 wt% Mn. A slight decrease is observed at 2 wt% Mn (345 F g^−1^), followed by a more pronounced drop at 5 wt% Mn (281 F g^−1^) and 10 wt% Mn (129 F g^−1^). The optimal KMnO_4_ percentage for 0.3 M NaCl is thus 1 wt%, where the highest specific capacitance is achieved.

A similar trend is observed, with specific capacitance increasing from 80 F g^−1^ (pristine AC, 0 wt% Mn) to a peak value of 452 F g^−1^ at 2 wt% Mn. A decrease follows, with 1 wt% Mn showing 425 F g^−1^, 0.7 wt% Mn at 404 F g^−1^, 5 wt% Mn at 287 F g^−1^ and 10 wt% Mn at 195 F g^−1^. The optimal KMnO_4_ percentage for 0.5 M NaCl is 2 wt%, providing the highest capacitance.

The incorporation of Mn enhances surface wettability, improving the interaction between the electrode and electrolyte [22,34]. Mn doping introduces functional groups (e.g. Mn–O, Mn–OH), facilitating enhanced charge storage via electric double-layer capacitance and mild pseudocapacitance. Mn species improve electrical conductivity, reducing internal resistance and increasing charge transfer efficiency. The porous structure remains accessible to ions, allowing efficient ion transport and charge storage.

At higher Mn content (greater than or equal to 5 wt%), excessive MnO*x* formation may block micropores, reducing the effective surface area available for ion adsorption. The electrical conductivity may decrease due to the insulating nature of excess Mn oxide, leading to higher charge transfer resistance. The increase in Mn oxide clusters might also lead to the agglomeration of particles, reducing electrolyte access to active sites.

Higher NaCl concentration (0.5 M) yields higher specific capacitance values overall. Increased electrolyte concentration enhances ionic conductivity, facilitating better charge accumulation and faster charge–discharge processes. The difference in optimal Mn content suggests that electrolyte concentration plays a role in determining the best doping level—lower concentrations (0.3 M) favour 1 wt% Mn, whereas higher concentrations (0.5 M) favour 2 wt% Mn. These findings confirm that controlled Mn incorporation can significantly improve capacitive performance for potential applications in energy storage and CDI.

Activated carbon is among the most widely used materials in both supercapacitor and CDI applications due to its high surface area, tunable porosity and chemical stability. Consequently, extensive research efforts have focused on synthesizing AC from various natural and industrial precursors, using different chemical activation agents to enhance its electrochemical properties, particularly specific capacitance. As summarized in table 1, the specific capacitance values of AC-based materials reported in the literature vary widely, ranging from 43 to 368 F g^−1^, depending on the source material, activation technique and electrode preparation method.

**Table 1.** Comparison of specific capacitance values of various activated carbon-based materials reported in literature and the present study.

catalyst	activation	electrolyte	sp. cap. (F g^−1^)	ref.
jute sticks	NaHCO_3_	glycerol-based bio-electrolyte	142	[35]
pinecone	KOH	1.0 M H_2_SO_4_	43	[36]
oil tea shell	ZnCl_2_	1.0 M KOH	146	[37]
waste coffee grounds	KOH	2.0 M KOH	105.3	[38]
banana fibre	ZnCl_2_	1.0 M Na_2_SO_4_	86	[39]
waste coffee beans	ZnCl_2_	1 M H_2_SO_4_	368	[40]
sugarcane bagasse	NaOH	1 M H_2_SO_4_	109	[41]
cherry stones	KOH	2 M H_2_SO_4_	174	[42]
sugarcane bagasse	ZnCl_2_	1 M H_2_SO_4_	300	[43]
cassava peel waste	KOH + CO_2_	0.5 M H_2_SO_4_	153	[44]
beer lees	KOH.	0.1M H_2_SO_4_	188	[45]
coffee endocarp	CO_2_+KOH	1M H_2_SO_4_	85	[46]
rice husk beet sugar	NaOH	1 M H_2_SO_4_	114	[47]
monoliths oil palm	KOH +CO_2_	1 M H_2_SO_4_	149	[48]
rice husks	NaOH	0.5 M K_2_SO_4_	198	[49]
coniferous-pine-biomass	KOH	1 M Na_2_SO_4_	90	[50]
peanut shell	KOH	1 M H_2_SO_4_	186	[51]
olive residues	H_3_PO_4_	1 M H_2_SO_4_	176	[52]
olive residues	H_3_PO_4_	1 M Na_2_SO_4_	172	[52]
nanoporous carbon	K_2_CO_3_	2.5 M KNO_3_	140	[53]
African maize cobs	H_2_SO_4_	1 M Na_2_SO_4_	78.5	[54]
olive tree	KOH	1 M H_2_SO_4_	180	[55]
rice husks	H_3_PO_4_	1 M H_2_SO_4_	88.5	[56]
commercial AC	~	0.3 M NaCl	62	this study
commercial AC	~	0.5 M NaCl	80	
Mn-doped AC	hydrothermal treatment	0.3 M NaCl	405	
Mn-doped AC		0.5 M NaCl	452	

In the present study, we employed commercial AC as a model and developed a novel and straightforward hydrothermal doping strategy using potassium permanganate to incorporate Mn species onto the AC surface. This method led to a significant improvement in specific capacitance, increasing from 62 to 405 F g^−1^ and from 80 to 452 F g^−1^ for 0.3 and 0.5 M NaCl solutions, respectively. The obtained specific capacitance is notably higher than the values reported for most other AC-based materials as shown in the table. The proposed strategy is not only effective but also highly versatile and can be applied to AC derived from various sources. This highlights the potential of Mn doping as a universal approach to boost the electrochemical performance of AC in CDI and related energy storage applications.

**Figure 9.**
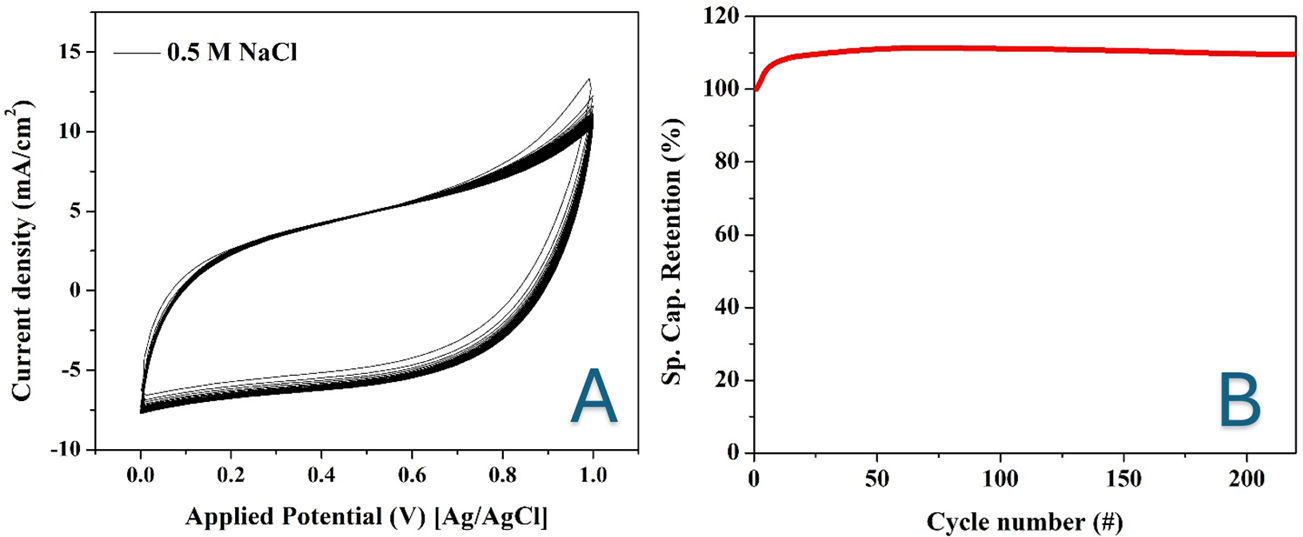
Cyclic voltammetry for the Mn-doped AC electrode (2 wt% sample) in 0.5 M NaCl for more than 200 cycles at scan rate of 100 mV s^−1^ (A) and specific capacitance retention during the applied cycles (B).

In our study, CV measurements were conducted over 200 successive cycles to evaluate the electrochemical stability of Mn-AC electrodes. The results, depicted in figure 9A,B, demonstrate that the CV curves remained consistent across all cycles, and the specific capacitance increased to approximately 112% of the initial value before stabilizing.

The observed increase in specific capacitance during the initial cycles can be attributed to an activation process commonly seen in electrochemical capacitors. This process involves several key factors [57]. Initially, the electrolyte gradually infiltrates the porous structure of the electrode material, enhancing the electrode–electrolyte interface. This improved contact allows more active sites to participate in charge storage, leading to increased capacitance. Similar behaviour has been reported in other studies, where the capacitance increased during early cycling due to better electrolyte access to the electrode material [58]. Repeated cycling can induce slight structural modifications within the electrode material, such as the rearrangement of Mn species or carbon matrices. These changes can create additional electrochemically active sites or more favourable pathways for ion transport, contributing to enhanced capacitance. For instance, studies have shown that Mn-doped electrodes undergo structural transformations during initial cycles, leading to improved electrochemical performance. The Mn doping introduces redox-active sites that can contribute to pseudocapacitance. During the initial cycles, these sites may become more accessible or active, further boosting the overall capacitance. Research indicates that the activation process in Mn-based electrodes can enhance pseudocapacitive contributions over time.

The stability of the CV curves over 200 cycles, along with the sustained increase in specific capacitance, underscores the robustness and durability of the Mn-AC electrodes. These findings suggest that the electrodes not only maintain structural integrity but also exhibit improved performance with continued use, making them promising candidates for practical supercapacitor applications.

#### 3.2.2. Capacitive deionization cell

**Figure 10.**
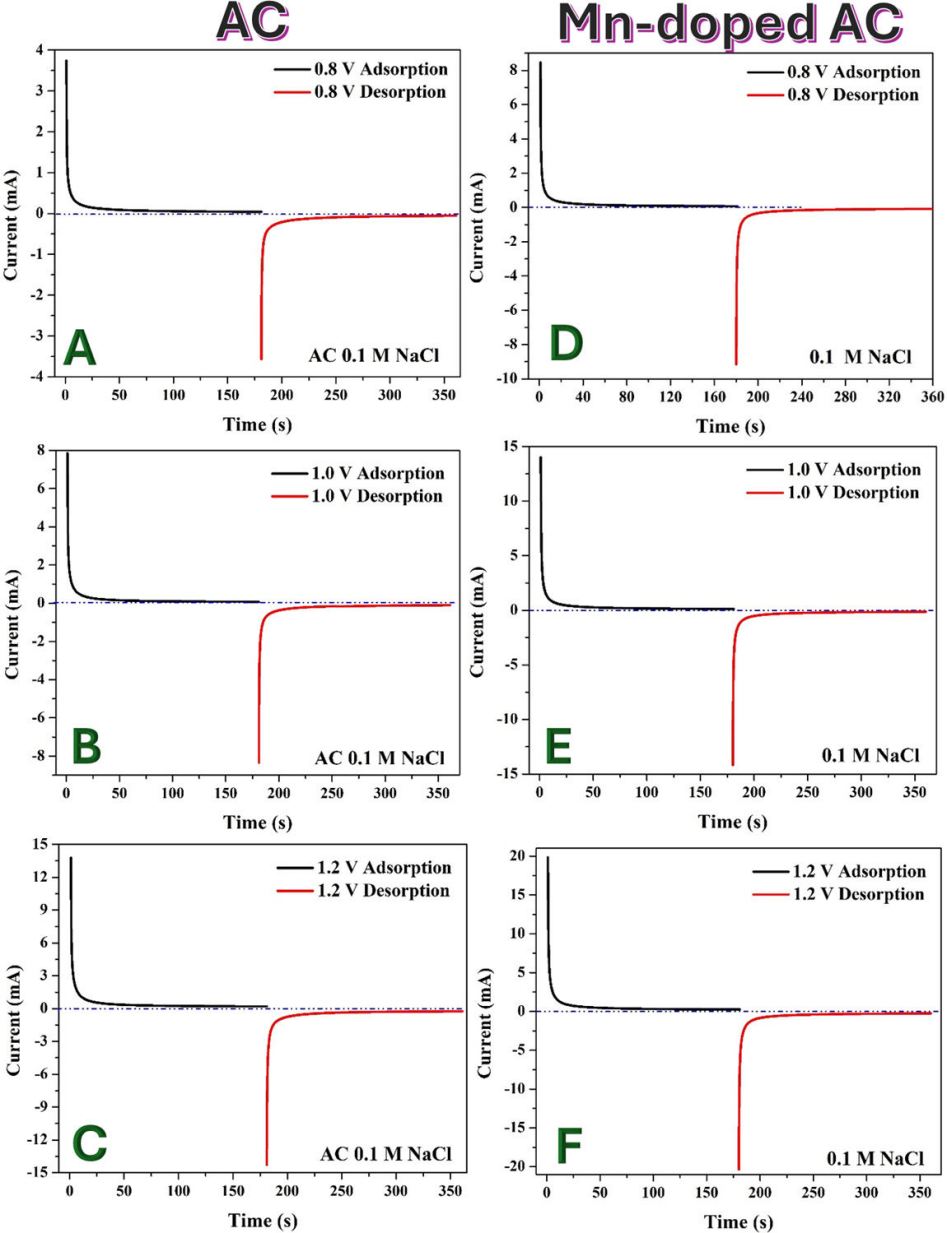
Chronoamperometry with switching polarity at different voltages (0.8, 1 and 1.2 V) for CDI cells using pristine (A–C) and Mn-doped (1 wt% sample) (D–F). AC electrodes using 0.1 M NaCl solution.

A CDI cell was assembled using electrodes fabricated from pristine AC and Mn-AC (1 wt%). The electrodes were separated by a 400 μm spacer, and the system was tested in batch mode using 0.1 M NaCl solution. The CDI performance was evaluated via chronoamperometry measurements at 0.8, 1.0 and 1.2 V, with polarity switching to study ion adsorption and desorption behaviour. The results are shown in figure 10.

Chronoamperometry is a valid and widely used technique for evaluating CDI cell performance because it directly measures the current response during ion electrosorption and desorption, providing insights into the ion removal rate and charge storage dynamics. The initial high current corresponds to the rapid adsorption of Na^+^ and Cl⁻ ions onto the electrode surfaces, followed by a decline as the adsorption sites become saturated and also due to mass transfer limitation. The desorption process, induced by reversing the voltage polarity, results in an observable current response, confirming the effective release of adsorbed ions. The polarity reversal effectively releases the adsorbed ions, demonstrating the regenerability of the electrodes and confirming CDI’s applicability for desalination.

For the pristine AC-based CDI cell, the starting current values at different applied voltages were: 0.8 V: 3.75 mA, 1.0 V: 7.84 mA and 1.2 V: 13.79 mA. The increasing starting current with voltage indicates that higher applied voltages enhance the driving force for ion electrosorption, leading to increased charge storage and a higher rate of ion removal. However, the sharp current decay suggests rapid saturation of active sites, which may limit long-term performance.

For the Mn-AC-based CDI cell, the starting current values were significantly higher: 0.8 V: 8.46 mA, 1.0 V: 14.01 mA and 1.2 V: 19.85 mA. The higher initial current for Mn-AC compared with pristine AC demonstrates the enhanced ion adsorption capability due to: (i) higher electrochemical activity: Mn doping introduces additional functional groups that improve charge transfer and increase available adsorption sites; (ii) improved wettability: the Mn-modified surface promotes better interaction with the electrolyte, facilitating faster ion adsorption kinetics; and (iii) increased capacitance: as shown in previous CV results, Mn-AC has a significantly higher specific capacitance, directly contributing to improved charge storage and ion adsorption efficiency.

Chronoamperometry results confirm that Mn-AC significantly improves CDI performance by enhancing charge storage, ion adsorption rate and cycling stability. The superior electrochemical properties of Mn-AC make it a promising electrode material for efficient brackish water desalination via CDI.

Electrosorption is a key parameter in assessing the efficiency of CDI electrodes. Chronoamperometry measurements provide the necessary data to estimate the amount of electrosorbed ions over time. This study compares the electrosorption capacity of prepared Mn-AC and pristine AC at three different applied voltages: 0.8, 1.0 and 1.2 V.

Electrosorption capacity is determined by integrating the current response over time, as it reflects the amount of charge transferred during ion adsorption. The electrosorption capacity **(***E_cp_*, in mg g^−1^**)** can be estimated using the following equation:


(3.3) $$E_{\text{cp}}=\frac{M}{zmF}\int I\left(t\right)\text{d}t,\;$$

where *M* is the molar mass of NaCl (58.44 g mol^−1^), *z* is the charge number (z = 1 for Na^+^ and Cl⁻), *F* is Faraday’s constant (96 485 C mol^−1^), *m* the mass of the active material in the electrode, *I(t)* is the measured current as a function of time, *∫I(t)*d*t* represents the total charge transferred (coulombs) during ion adsorption. This approach allows the estimation of the total ions removed from solution by the CDI cell during the adsorption process.

**Figure 11.**
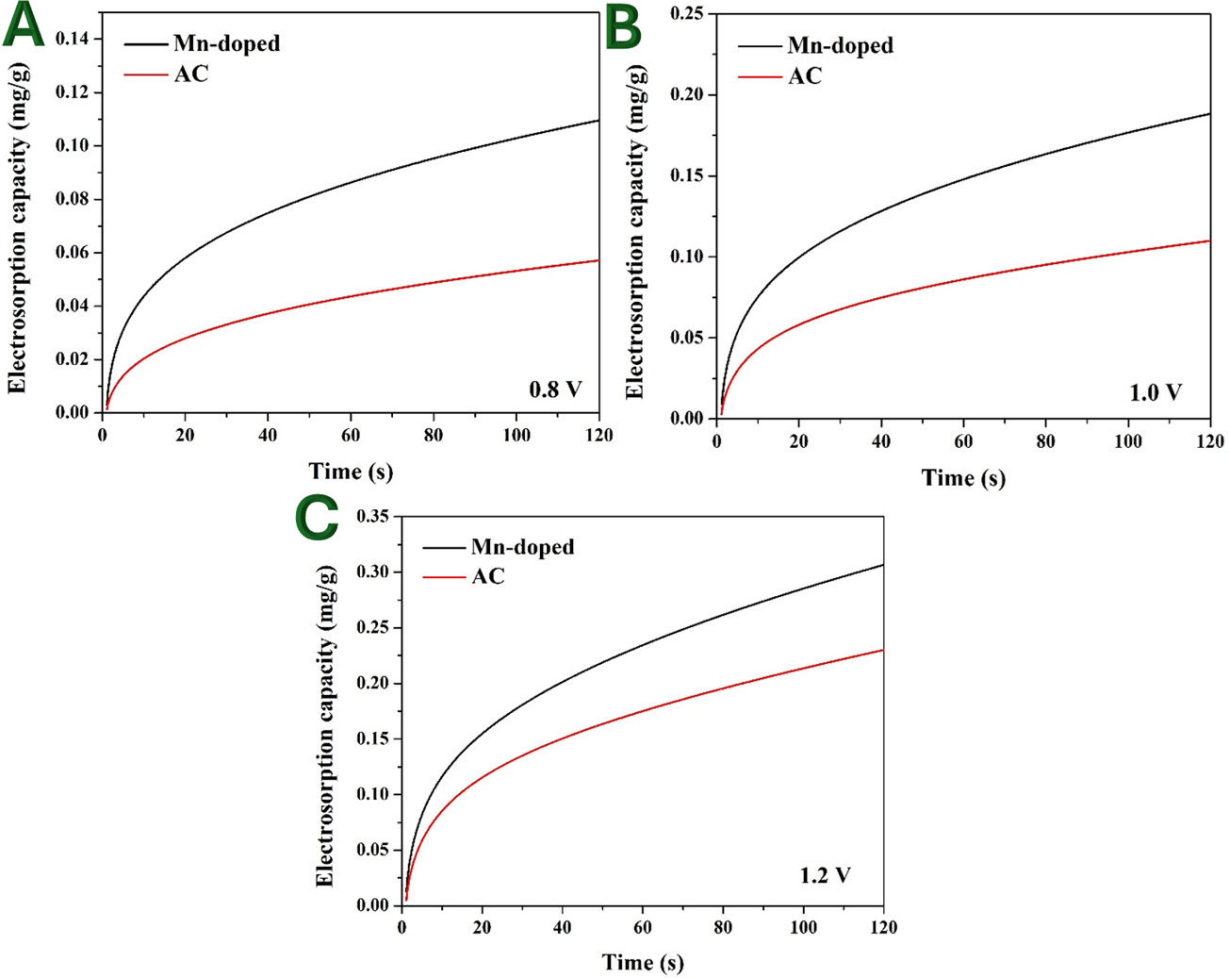
Electrosorption capacity of ions for CDI assembled with pristine and Mn-doped (2 wt% electrode) at 0.8 V (A), 1.0 V (B) and 1.2 V (C) using 0.1 M NaCl solution.

Figure 11A displays the electrosorption in the Mn-doped and pristine AC CDI cells at 0.8 V. As shown, a maximum electrosorption capacity was 0.11 and 0.06 mg g^−1^ for the doped and pristine AC electrode, respectively. Mn-AC shows nearly two times higher electrosorption capacity than pristine AC, indicating enhanced ion storage capability. The improved performance is attributed to Mn doping, which enhances electrochemical activity, pore accessibility and surface wettability. At lower voltages, the driving force for ion adsorption is relatively weak, but Mn doping facilitates better charge transfer and ion accommodation within the electrode.

At 1.0 V (figure 11B), the estimated electrosorption capacity was 0.19 and 0.11 mg g^−1^ for the treated and original AC electrode, respectively. As the applied voltage increases, the electric field strength enhances, promoting stronger electrosorption forces and improving ion removal efficiency. The difference between Mn-doped and pristine AC electrodes remains significant, showing that Mn incorporation provides a consistent advantage across different applied voltages. The increased electrosorption at 1.0 V compared with 0.8 V is due to higher charge accumulation, which allows more ions to be removed from the electrolyte.

In the same fashion, at 1.2 V (figure 11C), the doped and naked AC electrodes could achieve electrosorption capacity of 0.31 and 0.23 mg g^−1^, respectively. The highest applied voltage (1.2 V) leads to the highest electrosorption values for both electrodes. The increase in electric double-layer formation enhances ion attraction, leading to higher CDI efficiency. The electrosorption difference between Mn-doped and pristine AC decreases slightly at this voltage, possibly due to saturation effects or electrode polarization limitations at high potentials.

Chronoamperometry-derived electrosorption results confirm that Mn-AC significantly enhances ion adsorption capacity, making it a more efficient electrode material for CDI applications. The enhancement in electrosorption performance is attributed to higher surface wettability, improved conductivity and increased ion accessibility due to Mn incorporation. These findings further establish Mn-AC as a promising candidate for energy-efficient water desalination technologies.

**Figure 12.**
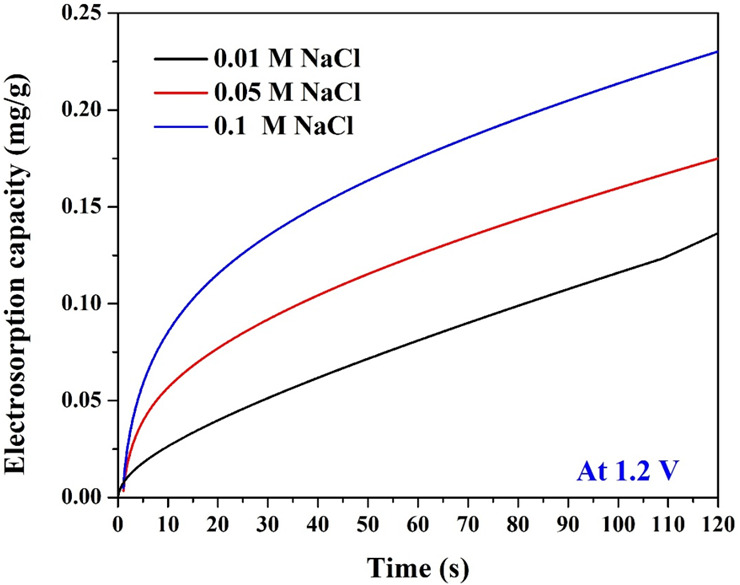
Effect of the NaCl solution concentration on the electrosorption capacity of the proposed Mn-doped AC (1 wt% sample) electrode at applied voltage of 1.2 V.

The influence of NaCl solution concentration on the electrosorption capacity was investigated at a fixed applied voltage of 1.2 V and 120 s. The results, presented in figure 12, demonstrate how varying electrolyte concentration affects ion adsorption efficiency in CDI using Mn-AC electrodes. The observed electrosorption trends are: 0.01 M NaCl: 0.14 mg g^−1^, 0.05 M NaCl: 0.17 mg g^−1^ and 0.1 M NaCl: 0.23 mg g^−1^.

The data indicate a clear increase in electrosorption capacity with rising NaCl concentration. This trend can be explained based on electrochemical and transport principles governing ion adsorption in CDI systems. In CDI, ion adsorption occurs due to the formation of an electric double layer at the electrode surface. When the NaCl concentration increases, more free Na^+^ and Cl⁻ ions are available in the solution, leading to higher ionic flux toward the electrode surface. At 0.1 M NaCl, the system provides maximum ion availability, resulting in the highest electrosorption capacity (0.23 mg g^−1^). At low concentrations (0.01 M), the number of available ions is limited, and the double-layer capacitance is lower, reducing charge storage and electrosorption. At moderate concentrations (0.05 M), the increased ion availability leads to better charge screening, improving electrosorption efficiency. At high concentrations (0.1 M), a denser ion layer forms at the electrode interface, further enhancing adsorption capacity.

A higher NaCl concentration increases the electrical conductivity of the solution, reducing the charge transfer resistance and allowing for more efficient electrosorption. Higher conductivity facilitates faster ion movement, leading to improved charge compensation at the electrode surface and increasing overall adsorption. These findings confirm that optimizing electrolyte concentration is crucial for maximizing CDI performance and ion removal efficiency.

**Figure 13.**
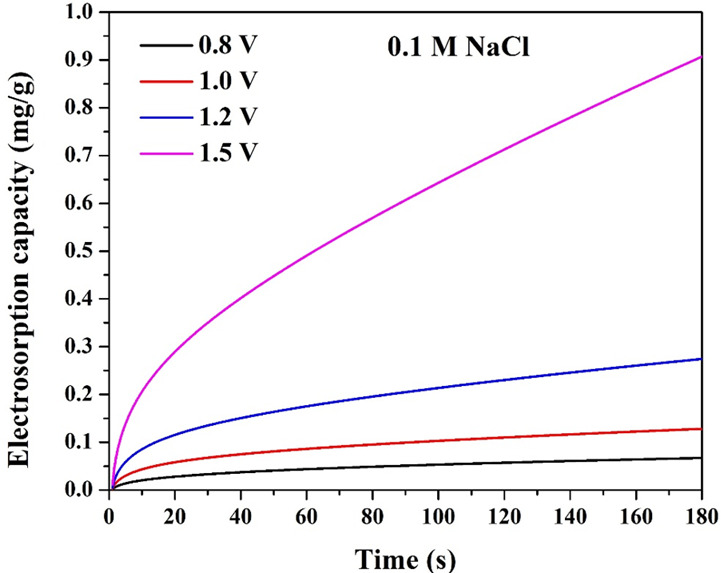
Effect of the applied voltage on the electrosorption capacity of the proposed Mn-doped AC (1 wt% sample) electrode at 0.1 M NaCl solution.

The impact of applied voltage on electrosorption capacity was evaluated at a fixed NaCl concentration of 0.1 M. The results, as shown in figure 13, demonstrate how increasing the applied voltage enhances ion adsorption efficiency in the CDI system. The electrosorption capacity was measured at 120 s for different applied voltages. The observed electrosorption capacity was 0.07, 0.13, 0.27 and 0.91 mg g^−1^ for 0.8, 1, 1.2 and 1.5 V, respectively. The data indicate a clear increase in electrosorption capacity with rising voltage, highlighting the role of electric field strength in ion removal efficiency.

In CDI, ions are adsorbed onto the electrode surface due to the formation of the electric double layer (EDL). Increasing the applied voltage enhances the electrostatic attraction force between the charged electrode surface and the Na^+^/Cl⁻ ions, leading to greater electrosorption. At higher voltages, the driving force for ion movement is stronger, allowing for more ions to be captured at the electrode interface. The increase in electrosorption is not linear with voltage. Instead, there is an accelerated enhancement in capacity at higher voltages. The sharp increase from 1.2 V (0.27 mg g^−1^) to 1.5 V (0.91 mg g^−1^) suggests that beyond a threshold voltage, a significant increase in charge storage and ion removal occurs. This nonlinearity is attributed to the saturation of adsorption sites at lower voltages, while higher voltages facilitate deep penetration of ions into micropores.

At moderate voltages (less than or equal to 1.2 V), the adsorption mechanism is primarily electrostatic (EDL formation). At higher voltages (greater than or equal to 1.5 V), Faradaic reactions may begin to occur, contributing to increased charge storage but potentially leading to side reactions (e.g. water splitting or electrode degradation). The significantly higher electrosorption at 1.5 V suggests that additional surface redox processes may enhance ion retention. The increasing electrosorption capacity with voltage increasing confirms that higher voltages enhance ion removal.

## 4. Conclusions

This study demonstrated the successful synthesis and application of Mn-AC as an efficient electrode material for CDI. Characterization results confirmed the successful incorporation of Mn into the carbon structure, enhancing its electrochemical properties. Electrochemical tests revealed that Mn-AC electrodes exhibited higher charge storage capacity and superior electrosorption performance compared with pristine AC. CDI performance evaluations showed that Mn doping significantly improved ion removal efficiency, with 1−2 wt% Mn-AC achieving the highest performance. The enhanced CDI efficiency was attributed to increased specific capacitance, improved wettability and better ion adsorption kinetics. Moreover, higher applied voltages and NaCl concentrations further enhanced electrosorption capacity. Overall, Mn-AC offers a scalable and cost-effective approach for improving CDI technology. Future research should focus on long-term stability, scalability and advanced electrode architectures to further enhance CDI efficiency and commercial viability.

## Data Availability

The data, code and material characterizations have been provided in electronic supplementary material [59].
